# Network analysis of depressive and anxiety symptoms in older Chinese adults with diabetes mellitus

**DOI:** 10.3389/fpsyt.2024.1328857

**Published:** 2024-01-29

**Authors:** Yajuan Zhang, Yi Cui, Yijun Li, Hongliang Lu, He Huang, Jiaru Sui, Zhihua Guo, Danmin Miao

**Affiliations:** ^1^ Department of Military Medical Psychology, Air Force Medical University, Xi’an, China; ^2^ Department of Nursing, Air Force Medical University, Xi’an, China

**Keywords:** depression, anxiety, network analysis, people with diabetes, older adults

## Abstract

**Background:**

The move away from investigating mental disorders as whole using sum scores to the analysis of symptom-level interactions using network analysis has provided new insights into comorbidities. The current study explored the dynamic interactions between depressive and anxiety symptoms in older Chinese adults with diabetes mellitus (DM) and identified central and bridge symptoms in the depression-anxiety network to provide potential targets for prevention and intervention for depression and anxiety.

**Methods:**

This study used a cross-sectional design with data from the 2017–2018 wave of the Chinese Longitudinal Healthy Longevity Survey (CLHLS). A regularized partial correlation network for depressive and anxiety symptoms was estimated based on self-reported scales completed by 1685 older adults with DM aged 65 years or older. Depressive and anxiety symptoms were assessed using the 10-item Center for Epidemiologic Studies Depression Scale (CESD-10) and the Seven-Item Generalized Anxiety Disorder Scale (GAD-7), respectively. Expected influence (EI) and bridge expected influence (BEI) indices were calculated for each symptom.

**Results:**

According to cutoff scores indicating the presence of depression and anxiety, the prevalences of depression and anxiety in our sample were 52.9% and 12.8%, respectively. The comorbidity rate of depression and anxiety was 11.5%. The six edges with the strongest regularized partial correlations were between symptoms from the same disorder. “Feeling blue/depressed”, “Nervousness or anxiety”, “Uncontrollable worry”, “Trouble relaxing”, and “Worry too much” had the highest EI values. “Nervousness or anxiety” and “Everything was an effort” exhibited the highest BEI values.

**Conclusion:**

Central and bridge symptoms were highlighted in this study. Targeting these symptoms may be effective in preventing the comorbidity of depressive and anxiety symptoms and facilitate interventions in older Chinese adults with DM who are at risk for or currently have depressive and anxiety symptoms.

## 1 Introduction

Diabetes mellitus (DM) is a metabolic disease characterized by hyperglycemia and caused by both genetic and environmental factors (1). The number of adults with DM worldwide is increasing rapidly, and according to the International Diabetes Federation (IDF) report, this number has currently reached 537 million and is expected to increase to 643 million by 2030 (2). China has the largest number of people with diabetes in the world, with about 140 million in 2021 (3). With the increasingly serious problem of population aging in China, the proportion of people over 65 years with diabetes is increasing, and older adults have become the primary demographic of people with diabetes (4). Furthermore, according to the guideline for the management of diabetes mellitus in older people in China (2021 edition), type 2 diabetes mellitus (T2DM) is predominant in the over 65-year-old Chinese population with diabetes, while type 1 diabetes mellitus (T1DM) occurs in the minority (5). In this study, however, diabetes refers to both type 1 and type 2 diabetes.

DM is a chronic non-communicable disease that seriously threatens mental health. The incidence of depression and anxiety disorders (assessed by the Composite International Diagnostic Interview) in people with diabetes is much higher than in the population at large over time, and is 60% higher for major depressive disorder and 123% for general anxiety disorder (6). In particular, the complex condition of older adults, the decline in their physical function and immune systems, long-term monitoring of blood glucose and diet control, and the increased economic pressure resulting from long-term drug treatment, together increase the susceptibility of older adults with DM to comorbid depression and anxiety (7, 8). DM interacts bidirectionally with depression and anxiety. On the one hand, as mentioned above, DM increases the prevalence of depression and anxiety; on the other hand, depression and anxiety can be independent risk factors for the occurrence and development of DM and are known to predict the incidence of later DM (9, 10).

The comorbidity of depression and anxiety is also common in older adults with diabetes. It has been reported that approximately 30% of those with major depressive disorder (MDD) and roughly 50% of those with general anxiety disorder (GAD) meet the criteria for a dual MDD/GAD diagnosis in a sample of people with diabetes with mean age of 57.8 years (6). In the general population, the presence of either depression or anxiety often increases the risk of developing the other (11), and the comorbidity of depression and anxiety results in more severe symptoms, fewer effective treatments are available, and the prognosis is poorer than for either disorder alone (12). Furthermore, people with diabetes with depression and anxiety also have an increased risk of diabetes complications, have a poor prognosis, poor blood glucose control and have lower quality of life (13–15). Therefore, the comorbidity of depression and anxiety in older adults with DM is an important research topic.

Most prior studies on comorbid depression and anxiety have been based on the assumption that anxiety and depression are holistic psychopathological constructs and have generally studied them at a disorder level, using the total score of the corresponding measurement scale to evaluate the severity of each disorder. However, such an approach ignores the interactions between individual symptoms (i.e., items of the measurement scales) and masks the heterogeneity of the various symptoms (16, 17). The pervasive use of sum-scores (i.e., summing the scores for each item) has hampered progress in key research fields such as the search for more effective intervention targets for anxiety and depression (18). Therefore, to better understand the comorbidity of depression and anxiety in older adults with DM and identify possible targets for interventions, we need to adopt a more fine-grained research methodology such as the analysis of individual symptoms and their interactions. Notably, in this study, unless otherwise stated, the term “symptom” refers to items from the scales rather than clinical diagnoses.

Network analysis is an emerging, data-driven approach that provides a new perspective for understanding psychopathology and comorbidity. It permits the structure of mental disorders and the interactions between individual symptoms to be investigated and visualized (19–21). Network analysis is based on the assumption that psychiatric disorders emerge from active interactions between various symptoms, and different symptoms may actively reinforce or inhibit other symptoms, rather than simply viewing symptoms as reflecting underlying latent variables (20, 21). The high comorbidity between depression and anxiety means that the specific symptoms of one psychiatric disorder will increase the risk of developing the other. It is both reasonable and feasible, therefore, to regard them as a complex network comprising the interactions of different symptoms (22, 23). Network analysis helps identify relatively important relationships between the individual symptoms of anxiety and depression. A centrality index can be calculated to quantify the influence of individual symptoms in the network, and determine critical central symptoms that are more likely to activate other symptoms and play major roles in the onset and/or maintenance of the mental disorder (24). Network analysis also calculates a bridge centrality index to identify important bridge symptoms that can facilitate the contagion of one disorder to another, leading to the development and maintenance of comorbidity (25).

Prior studies have used network analysis to explore comorbid symptom networks of anxiety and depression in different populations, such as people with epilepsy (26, 27), older people with functional impairment (28), nursing students (29, 30), people with MDD (31), and people with anxiety disorders (32, 33). However, the results of those studies have been inconsistent. One study based on network analysis examined diabetes distress and depressive and anxiety symptoms in middle-aged Canadians, and findings revealed strong connections between the anxiety symptom of “trouble relaxing” and the depressive symptom of “sleep problems,” as well as between the anxiety symptom of “restless” and the depressive symptom of “psychomotor agitation/retardation” (34). However, to date, depressive and anxiety symptoms in older Chinese adults with DM have not been studied using network analysis. Thus, despite the high prevalence of comorbid depression and anxiety in older adults with diabetes, which seriously affects their mental health and quality of life, these comorbid psychiatric disorders have not received due attention. Considering the data-driven nature of network analysis, the examination of different study populations with various symptoms of depression and anxiety can lead to heterogeneous results. Additionally, the features of symptoms are influenced by sociocultural factors, which can result in variations across countries. For example, culture impacts the experience of depression symptoms and depression is highly stigmatized in some cultures (35), while traditional Chinese social and cultural factors have been reported to potentially serve as protective factors against depression (36). Hence, findings based on other samples are not necessarily applicable to older Chinese adults with DM, and studies are warranted to investigate the comorbidity of depression and anxiety in this population.

The current study is the first to use network analysis to construct a symptom-level network of depression and anxiety in older Chinese adults with DM. We aimed to explore the dynamic interrelationships between depressive and anxiety symptoms. We also aimed to identify central symptoms and bridge symptoms to identify potential targets for the prevention and intervention of anxiety and depression in older Chinese adults with DM.

## 2 Materials and methods

### 2.1 Study design and participants

This study used a cross-sectional design based on data from the 2017–2018 wave of the Chinese Longitudinal Healthy Longevity Survey (CLHLS). The CLHLS is a nationally representative, population-based, ongoing survey focusing on older adults in mainland China. Following the baseline survey in 1998, the CLHLS has conducted seven waves of serial follow-up surveys in 2000, 2002, 2005, 2008–2009, 2011–2012, 2014, and 2017–2018. Due to the need for a representative sample, the CLHLS adopted a multi-stage disproportionate and targeted random sampling method (37). The participants were older adults (aged 65 years and above) and their children (aged 35–64 years) selected from about half of the counties and cities in 23 provinces, municipalities, and autonomous regions across China. More details about the CLHLS can be found elsewhere (38–40). The 2017–2018 wave of the CLHLS included 15,874 older adults aged 65 years and over (41). Following the example of previous studies (42, 43), older adults were defined in this study as those aged more than 65 years old. The inclusion criteria for this study were (1): validated age ≥ 65 (2), DM diagnosed by a hospital, and (3) complete data for all depressive and anxiety items. Participants with missing values or answers of “not able to answer”, “don’t know”, or “not applicable” in any scale items of interest were excluded. We focused on the general population of older adults with DM with a full range of symptom severity levels of depression and anxiety, such as ranging from “not depressed” to “severely depressed” rather than a clinical sample with formal diagnoses of depression and/or anxiety. Therefore, there were no other eligibility requirements for participants in this study. Finally, a total of 1685 older adults were included in the current study. The gender distribution of the included participants did not differ significantly from those excluded. However, there was a significant difference in age between included participants versus those excluded (*p* < 0.001). All methods were carried out in accordance with the Declaration of Helsinki and with relevant guidelines and regulations. Written informed consent was obtained from all participants included in this study. The ethical approvals of the CLHLS study were obtained from the Biomedical Ethics Committee, Peking University (IRB00001052–13074) and the Institutional Review Board, Duke University (Pro00062871). The CLHLS dataset used in this study is open, public, and free.

### 2.2 Measures

#### 2.2.1 10-item center for epidemiologic studies depression scale

The CESD-10 is a self-report scale used to measure how often each symptom of depression occurred during the past week and has been validated in Chinese older adults (44, 45). It comprises 10 items, each of which is rated using a 4-point Likert-type scale ranging from 0 = *never* to 3 = *always* (46). The total score on the CESD-10 can range from 0 to 30, with higher scores representing more severe symptoms of depression. In accordance with previous studies (45–47), a cutoff score of 10 was used to indicate the possible presence of depression. The Cronbach’s α coefficient of this scale was 0.79 in the current study, indicating good internal consistency.

#### 2.2.2 Seven-item generalized anxiety disorder scale

The GAD-7 is a reliable self-report scale used to assess the frequency of the most important diagnostic symptoms of GAD over the previous two weeks (48). It comprises seven items, each of which is rated using a 4-point Likert-type scale ranging from 0 = *not at all* to 3 = *nearly every day*. The total score for the GAD-7 can range from 0 to 21 with higher scores indicating more severe anxiety symptoms. Cutoff scores of 5, 10, and 15 represent mild, moderate, and severe levels of anxiety (48, 49). The Cronbach’s α coefficient of the GAD-7 was 0.91 in our sample, indicating excellent internal consistency.

### 2.3 Statistical analysis

#### 2.3.1 Network estimation

The program RStudio (version 4.3.1) was used to construct the network structure and calculate the expected influence (EI) and bridge expected influence (BEI) of each node. The R package *qgraph* was used to build and visualize the depression-anxiety network (50). The network was estimated via the Gaussian graphical model (GGM), which is an undirected network (51). According to a tutorial (52), we constructed the network based on Spearman correlations instead of polychoric correlations because of the ordinal nature of item scores, possibly skewed data distribution, and low frequency cross tables leading to biased polychoric correlations, as in previous studies (53, 54). In the constructed network, nodes represented symptoms and were divided into the depression community and the anxiety community; each edge represented the partial correlation between two nodes, with the confounding effects of all other nodes in the network eliminated by statistical controls (55). To regularize the network, a combination of least absolute shrinkage and selection operator (LASSO) and the extended Bayesian information criterion (EBIC) was adopted to shrink all the edges and attenuate small correlations to zero (52, 56, 57). We set the EBIC hyperparameter to 0.5 to determine the optimal network model, thereby creating a sparse and interpretable network (52, 56).

#### 2.3.2 Centrality and bridge centrality estimation

The R packages *qgraph* and *networktools* were used to calculate the centrality index (i.e., EI) and bridge centrality index (i.e., BEI) of each node to determine important central and bridge nodes, respectively (25, 50). The EI index was chosen because it outperforms other centrality indices when networks contain both positive and negative edges (58, 59). Node EI is the sum of non-absolute weights of all edges directly connected to a given node (58). Compared with other centrality measures such as node strength, the sum of the absolute value of its connections with other nodes in the network, EI can distinguish between positive and negative edges, and the signs of edge weights are important when assessing the nature and strength of a node’s cumulative influence within the network (e.g., the overall role of activating or remission effect on other nodes) (58). A higher EI value indicates the node is more positively associated with other nodes and exerts more influence on the entire network. Node BEI is the sum of the non-absolute weights of all edges directly linking a given node to nodes in another community, differing from bridge strength which sums the absolute values of the weights, and thus this index is the better option for networks containing both negative and positive edges (25). A higher BEI value suggests the node might contribute more to comorbidity and presents a higher risk of contagion from the current community to another community.

#### 2.3.3 Network accuracy and stability estimation

R package *bootnet* was used to estimate the robustness of the network by testing the accuracy of edge weights and the stability of the centrality and bridge centrality indices (55), to ensure the accuracy and replicability of the network analysis. The accuracy of the edge weights was assessed by computing 95% confidence intervals (CIs) using non-parametric bootstrapping (1000 bootstrapped samples); the narrower the 95% CI for each edge weight, the more accurate the edge weight estimation (60). The bootstrapped difference tests (α = 0.05, 1000 bootstrapped samples) were conducted to evaluate the differences between the edge weights of node pairs based on 95% CIs, with two edges being statistically different if zero was not included in the CI of the difference between the two edges (60, 61). The stabilities of EI and BEI were assessed by case-dropping bootstrapping (1000 bootstrapped samples) (55). We quantified stability using the correlation stability coefficient (CS-coefficient). The CS-coefficient should not be less than 0.25 and should preferably be greater than 0.5, which represents ideal stability (55). Subsequently, the differences between two node EIs or two node BEIs were also tested by bootstrapped difference tests (α = 0.05, 1000 bootstrapped samples) based on 95% CIs. Similarly, the two EIs or two BEIs were considered significantly different if zero was not included (55, 60).

## 3 Results

### 3.1 Descriptive statistics

The mean age of the included participants (N = 1,685) was 80.53 ± 9.98 years (mean ± standard deviation, range = 65–109 years); of whom 748 (44.4%) were male, 758 (45.0%) were registered urban residents, 648 (38.5%) resided in the city area, 872 (51.8%) were married and living with their spouses, 957 (56.8%) slept less than 7 h each day, 1092 (64.8%) were using antidiabetic medications, and 232 (13.8%) perceived diabetes to affect daily life rather seriously. The average years of education was 4.96 with the means of all non-missing values used to impute the missing values. The demographic characteristics of the sample are shown in **Table 1**. The prevalences of depression (defined as CESD-10 total score ≥ 10) and anxiety (defined as GAD-7 total score ≥ 5) in the present sample were 52.9% and 12.8%, respectively. Additionally, the comorbidity rate of depression and anxiety was 11.5%. **Table 2** shows the abbreviations, mean scores, standard deviations, EIs (raw values), and BEIs (raw values) for each symptom of depression and anxiety in the present network.

**Table 1:** Demographic characteristics of the sample (N = 1,685).

Variable	Mean (SD) or n (%)
**Age (years)**	80.53 (9.98)
Sex	
Male	748 (44.4%)
Female	937 (55.6%)
Hukou	
Registered urban residents	758 (45.0%)
Registered rural residents	924 (54.8%)
Missing	3 (0.2%)
Current residence region	
City	648 (38.5%)
Town	465 (27.6%)
Rural	572 (33.9%)
**Education (years)**	4.96 (4.69)
Ethnicity	
Han	1475 (87.5%)
Hui	13 (0.8%)
Zhuang	30 (1.8%)
Yao	1 (0.1%)
Man	6 (0.4%)
Others	4 (0.2%)
Missing	156 (9.3%)
Current marital status	
Currently married and living with spouse	872 (51.8%)
Separated	27 (1.6%)
Divorced	7 (0.4%)
Widowed	754 (44.7%)
Never married	11 (0.7%)
Missing	14 (0.8%)
Sleep duration each day	
≤7 h	957 (56.8%)
≥8 h	718 (42.6%)
Missing	10 (0.6%)
Smoking or not at present	
Yes	204 (12.1%)
No	1466 (87%)
Missing	15 (0.9%)
Drinking or not at present	
Yes	189 (11.2%)
No	1467 (87.1%)
Missing	29 (1.7%)
Exercising or not at present?	
Yes	675 (40%)
No	987 (58.6%)
Missing	23 (1.4%)
Whether to use the antidiabetic medications	
Yes	1092 (64.8%)
No	570 (33.8%)
Missing	23 (1.4%)
Whether diabetes affects daily life	
Rather serious	232 (13.8%)
More or less	715 (42.4%)
No	712 (42.3%)
Missing	26 (1.5%)

**Table 2:** Abbreviations, mean scores, standard deviations, EIs (raw values), and BEIs (raw values) for each symptom in the depression-anxiety network.

Symptoms	Abb	M	SD	EI	BEI
Depression symptoms (CESD-10)					
Feeling bothered	CESD1	0.79	0.58	0.90	0.05
Difficulty with concentrating	CESD2	1.02	0.70	0.68	0.06
Feeling blue/depressed	CESD3	0.73	0.58	1.12	0.09
Everything was an effort	CESD4	1.06	0.73	0.85	0.13
Hopelessness	CESD5	1.29	0.88	0.66	0.05
Feeling nervous/fearful	CESD6	0.65	0.60	0.84	0.08
Lack of happiness	CESD7	1.45	0.92	0.71	0.02
Loneliness	CESD8	0.64	0.65	0.92	0.04
Inability to get going	CESD9	0.50	0.60	0.90	0.10
Sleep disturbances	CESD10	1.36	0.77	0.39	0.13
Anxiety symptoms (GAD-7)					
Nervousness or anxiety	GAD1	0.32	0.56	1.05	0.38
Uncontrollable worry	GAD2	0.23	0.51	1.05	0.05
Worry too much	GAD3	0.28	0.54	1.02	0.11
Trouble relaxing	GAD4	0.20	0.47	1.04	0.09
Restlessness	GAD5	0.16	0.42	0.92	0.01
Easily annoyed/irritated	GAD6	0.20	0.47	0.89	0.09
Afraid something terrible might happen	GAD7	0.12	0.38	0.65	0.03

Abb, abbreviation; M, mean; SD, standard deviation; EI, expected influence; BEI, bridge expected influence; CESD-10, 10-item Center for Epidemiologic Studies Depression Scale; GAD-7, seven-item Generalized Anxiety Disorder scale.

### 3.2 Network structure

**Figure 1:**
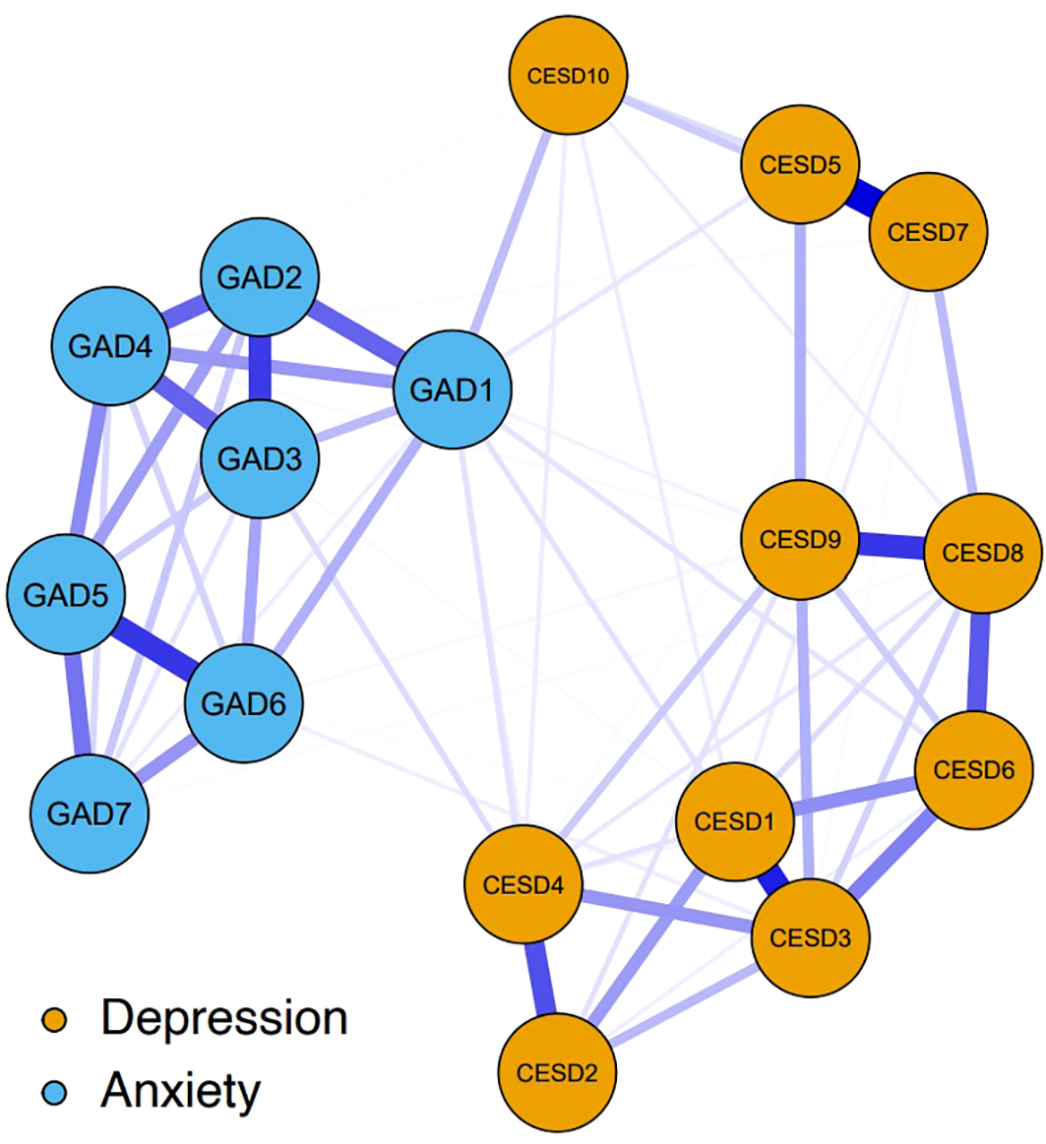
The network structure of depression and anxiety symptoms. The yellow nodes denote the depression symptoms (10-item Center for Epidemiologic Studies Depression Scale, CESD-10); the blue nodes denote the anxiety symptoms (seven-item Generalized Anxiety Disorder scale, GAD-7). The specific meanings of each node are shown in **Table 1**. Blue edges represent positive relations, with thicker and more saturated edges denoting stronger connections between symptom nodes. Nodes with stronger connections are closer to each other. The weights of the edges are provided in **Supplementary Table 1**.

**Figure 1** shows the network structure of depression and anxiety symptoms. The network comprised 17 nodes and was estimated with 62.5% (85 of 136) non-zero edges. All edges had positive weights. The six strongest edges that exhibited relatively strong regularized partial correlations were identified. Four of these were in the depression community, those being the edges between CESD2 “Difficulty with concentrating” and CESD4 “Everything was an effort” (weight = 0.26), between CSED8 “Loneliness” and CSED9 “Inability to get going” (weight = 0.30), between CSED1 “Feeling bothered” and CSED3 “Feeling blue/depressed” (weight = 0.33), and between CSED5 “Hopelessness” and CSED7 “Lack of happiness” (weight = 0.39). The other two were in the anxiety community, those being the edges between GAD2 “Uncontrollable worry” and GAD3 “Worry too much” (weight = 0.29) and between GAD5 “Restlessness” and GAD6 “Easily annoyed/irritated” (weight = 0.30). Although weaker, there were several edges linking depression nodes and anxiety nodes, hereafter referred to as bridge edges. Notably, we enumerated these bridge edges based on the relative size of edge weights rather than using a cutoff value or statistical comparisons using bootstrapped difference tests. GAD1 “Nervousness or anxiety” was positively connected to: CESD10 “Sleep disturbances” (weight = 0.10), CESD3 “Feeling blue/depressed”, CESD4 “Everything was an effort”, CESD5 “Hopelessness”, and CESD6 “Feeling nervous/fearful” (weights = 0.05 from CESD3 to CESD6). CESD4 “Everything was an effort” was also positively associated with GAD3 “Worry too much” (weight = 0.06). **Supplementary Table 1** gives all the edge weights within the depression-anxiety network.

### 3.3 Central symptoms and bridge symptoms

**Figure 2:**
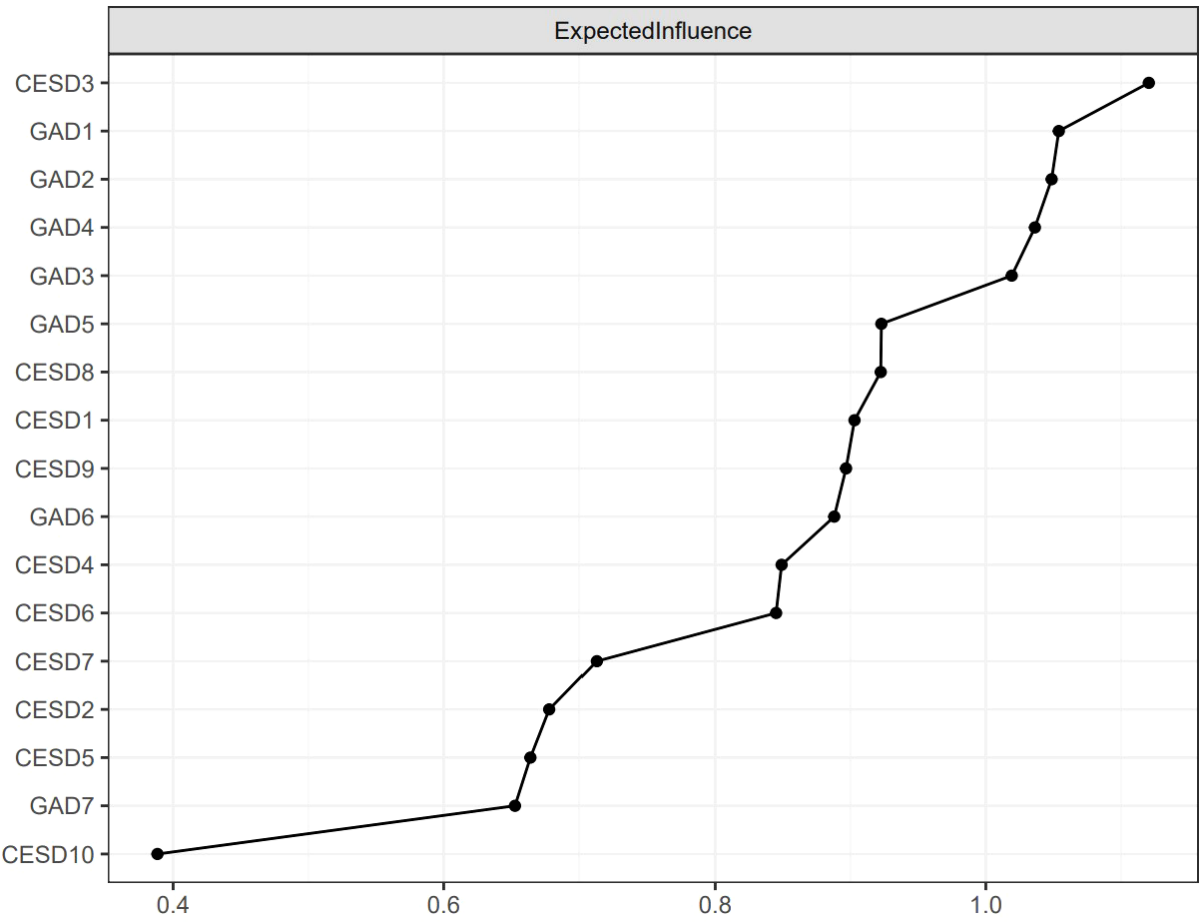
The raw values of EI for each node in the present network. The specific meanings of each node are shown in **Table 1**.

**Figure 3:**
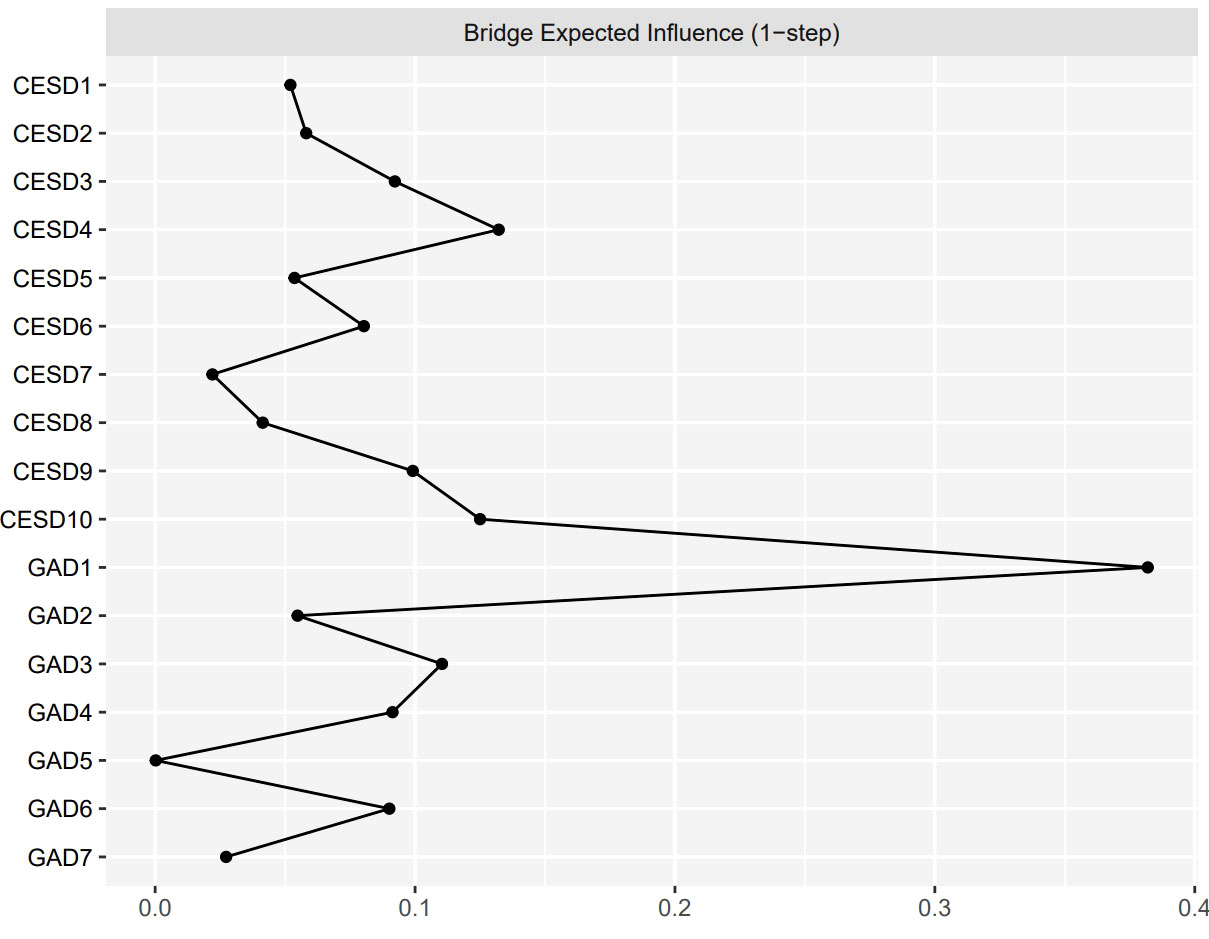
The raw values of BEI for each node in the present network. The specific meanings of each node are shown in **Table 1**.

**Figure 2** shows the EI indices of each node to assess their relative importance in the network. The five nodes with the highest EIs were CESD3 “Feeling blue/depressed” (EI = 1.12), GAD1 “Nervousness or anxiety” (EI = 1.05), GAD2 “Uncontrollable worry” (EI = 1.05), GAD4 “Trouble relaxing” (EI = 1.04), and GAD3 “Worry too much” (EI = 1.02), indicating that those were the most influential symptoms. **Figure 3** shows the raw BEI values of each node. The node GAD1 “Nervousness or anxiety” and the node CESD4 “Everything was an effort” had the highest BEI values overall (BEI = 0.38 and 0.13, respectively), with the node GAD1 “Nervousness or anxiety” having the highest BEI by far. This indicates that these two nodes represent critical bridge symptoms.

### 3.4 Network accuracy and stability

**Figure 4:**
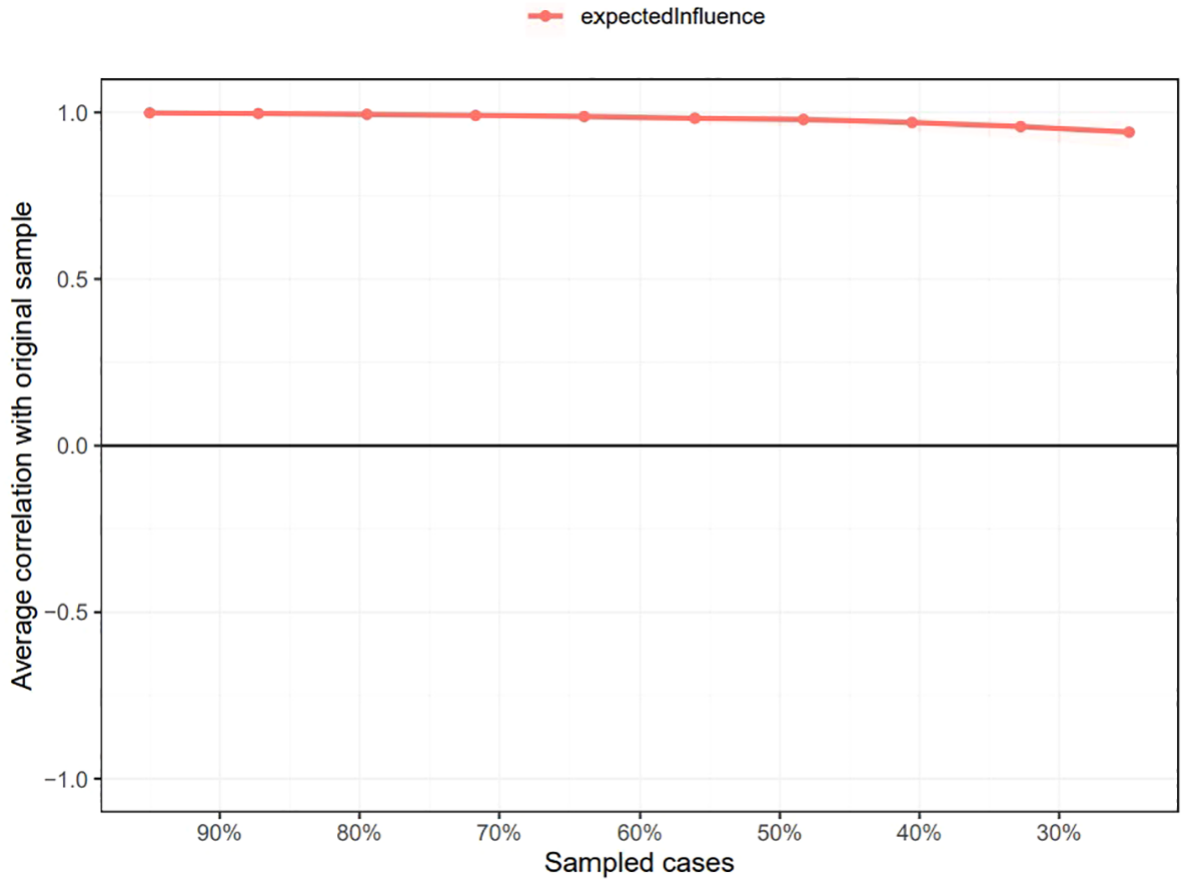
Stability of node expected influences in the network. The red bar represents the average correlation between node expected influences in the full sample and subsample with the red area depicting the 2.5th to the 97.5th quantile.

**Figure 5:**
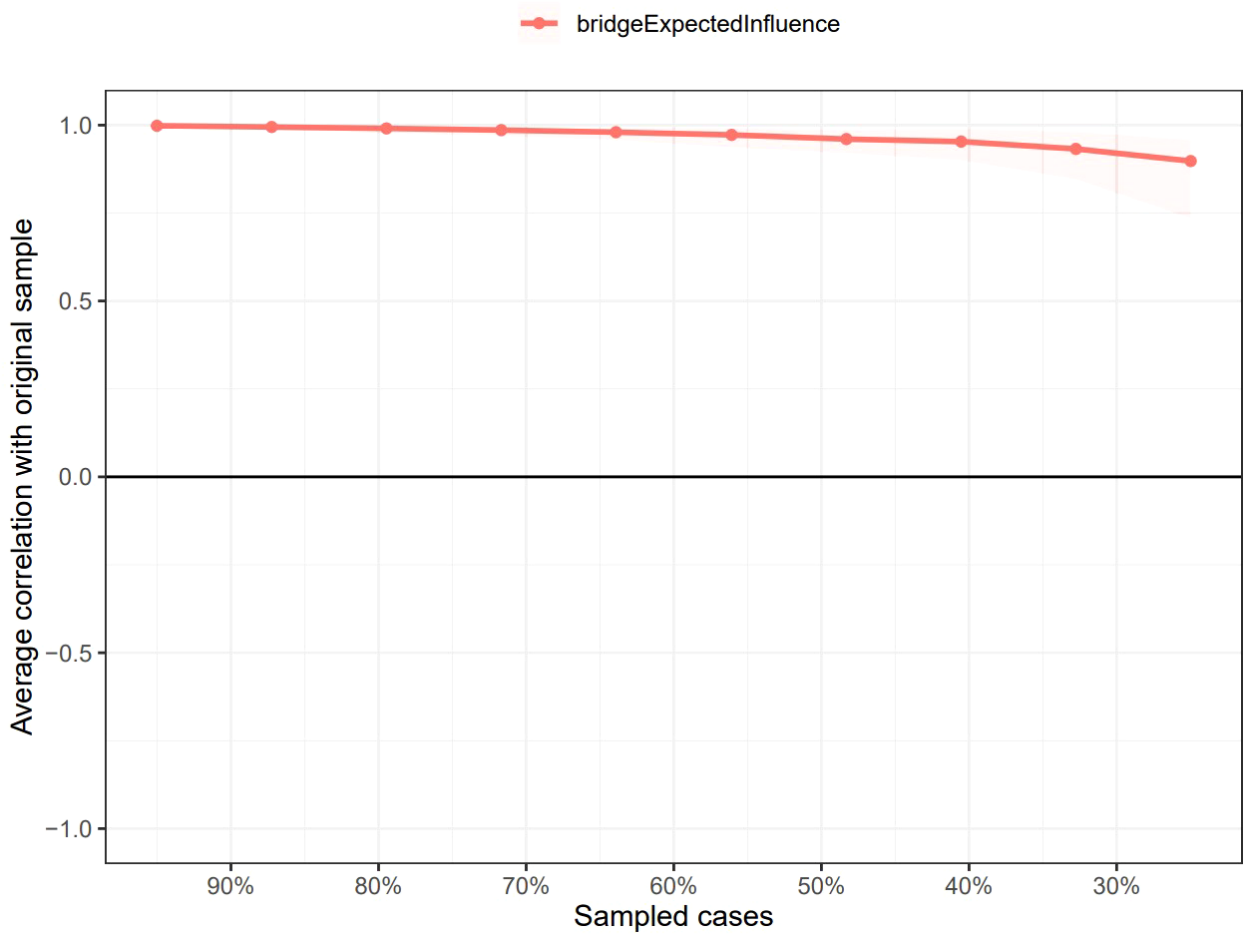
Stability of node bridge expected influences in the network. The red bar represents the average correlation between node bridge expected influences in the full sample and subsample with the red area depicting the 2.5th to the 97.5th quantile.

As shown in **Supplementary Figure 1**, the bootstrapped 95% CI was narrow, suggesting that the estimation of edge weights was accurate and stable. **Supplementary Figure 2** presents the bootstrapped difference test results for the edge weights, indicating that the weights of the six strongest edges were significantly higher than those of 88.1% - 98.8% of the other nodes. The CS-coefficients of EI and BEI were both 0.75, suggesting that the estimations of EI and BEI were both adequately stable (see **Figures 4**, **5**). The bootstrapped difference test for node EIs showed that the EI values of the five central nodes were significantly higher than those of 68.8% - 75% of the other nodes (see **Supplementary Figure 3**). **Supplementary Figure 4** illustrates the bootstrapped difference test for node BEIs, indicating that the BEI values of the two bridge nodes were significantly higher than those of 56.3% - 100% of other nodes.

## 4 Discussion

To the best of our knowledge, this is the first study to use network analysis to investigate symptom-level interactions between depression and anxiety in a group of older Chinese adults with DM. We found some important connections between individual symptoms. We also identified several influential central and bridge symptoms. These findings may facilitate our understanding of the dynamic interplay of individual symptoms in depression and anxiety, shed light on the pathological mechanisms that underly the development and maintenance of comorbid depression and anxiety, and provide better insights into potential intervention and treatment strategies.

The strongest edges appeared within each mental disorder community rather than in connections between the depressive and anxiety symptom communities. This is consistent with many previous studies that have used network analysis to examine the comorbidity of depression and anxiety and found that the strongest edges were between symptoms from the same disorder (26, 29, 31, 62–65), although they have used different assessment tools. The majority of previous studies have used the nine-item Patient Health Questionnaire (PHQ-9) and the GAD-7 to assess depressive and anxiety symptoms, respectively (26, 29, 62–65), except for Park and Kim’s study which used the Beck Depression Inventory and Beck Anxiety Inventory (31). The results of the present study identified four strong connections within the depressive community. Our findings are partly consistent with a prior study that used network analysis to examine insomnia and depressive symptoms (measured by the CESD-10), with the strong edges identified within the depression community of symptoms, i.e., “Loneliness”-”Inability to get going”, “Feeling bothered”-”Feeling blue/depressed”, and “Hopelessness”-”Lack of happiness” (66). Additionally, within the anxiety community, the finding that strong edges existed between GAD2 “Uncontrollable worry” and GAD3 “Worry too much” and between GAD5 “Restlessness” and GAD6 “Easily annoyed/irritated” is consistent with prior network analysis studies of comorbid depressive and anxiety symptoms assessed using the PHQ-9 and GAD-7, respectively (29, 62, 64). Together, these findings that the strongest edges existing within each community were expected because, from a theoretical perspective, the associated symptoms from the same community (e.g., depressive symptoms) interact closely with each other to induce mental disorders (e.g., depression) (20).

According to the network theory of psychopathology, EI may be a crucial way to identify influential central symptoms. Nodes with high EI are thought to be critical central symptoms that contribute to the development and maintenance of mental disorders (20, 58). By activating other symptoms in the network, these central symptoms are thought to trigger and maintain the other symptoms and, by extension, the psychopathological networks. The results of this study showed that CESD-3 “Feeling blue/depressed” was the symptom highest in EI, indicating its central role in the depression-anxiety network for Chinese older adults with DM. Similarly, “Depressed or sad mood” was found to be a central symptom, as has been previously reported in network analyses of depressive and anxiety symptoms in adolescents (67), psychiatric samples (mood, anxiety, personality, and psychotic disorders) (62), nursing students (30), and people diagnosed with both depression and anxiety disorders (68). The majority of these studies used PHQ-9 and GAD-7 to measure depressive and anxiety symptoms, respectively (62, 67, 68), except for Bai et al.’s study which used two-item PHQ and GAD-7 (30). Our findings are also consistent with other network analysis studies of individuals with elevated depressive symptoms and shift workers (with depressive symptoms assessed using the Inventory of Depressive Symptomatology and CESD-10, respectively) that reported that feeling depressed was one of the central symptoms (17, 66). Therefore, the central symptom of “Feeling blue/depressed” may be consistent across different populations; nonetheless, it should be examined further. Additionally, depressed or sad mood has been reported to be important for the prediction of MDD and increases the incidence of MDD (69), which is in line with our finding. Furthermore, prior studies have revealed that DM is associated with an increased risk of incident depressed mood in 70-to 79-year-old adults with DM (70). These lines of evidence support our finding that “Feeling blue/depressed” is critical to the development and maintenance of the depression-anxiety network in older adults with DM.

The symptom GAD2 “Uncontrollable worry” was another predominant central symptom (the second highest in EI overall) that emerged in the depression-anxiety network, suggesting that it may also contribute to the activation of other symptoms and the maintenance of the depression-anxiety network in older adults with DM. This is consistent with prior studies that have identified “Uncontrollable worry” as a central node in the network of depression and anxiety symptoms (measured by the PHQ-9 and GAD-7, respectively) in different populations (college students, patients diagnosed with both depression and an anxiety disorder, clinicians) based on strength centrality (65), strength and EI centrality (68), or EI centrality (64), respectively. In addition, we also found that GAD1 “Nervousness or anxiety”, GAD4 “Trouble relaxing”, and GAD3 “Worry too much” were high in EI and were thus identified as central symptoms. These findings are partially consistent with prior studies that have shown that “Trouble relaxing” (or “Unable to relax”) and “Excessive worry” (or “Too much worry”) had high centrality indices for the populations studied (27, 62, 64, 65, 68). The aforementioned studies all used the PHQ-9 and the GAD-7 to assess depressive and anxiety symptoms except for Gauld et al. (27) who used the Neurological Disorders Depression Inventory for Epilepsy (NDDI-E) and the GAD-7. However, GAD1 “Nervousness or anxiety” was not found to be a central symptom in prior studies. This inconsistency may be the result of the use of different study samples, e.g., older adults with DM in our study versus a psychiatric sample in Beard et al.’s study (62), people with epilepsy in Gauld et al.’s study (27), and people with both depression and an anxiety disorder in Kaiser et al.’s study (68). The characteristics of older adults with DM differ from those of individuals with neurological or psychiatric disorders. Serious diabetes complications such as macrovascular complications (e.g., cardiovascular disease) and microvascular complications (e.g., kidney disease and diabetic retinopathy and neuropathy) (71), as well as diabetes-related distress such as hypoglycemia induced by insulin treatment (72, 73), are all intractable problems for people with DM. In particular, the potential pathophysiology of DM in older adults is worse because of the adverse effects of aging on metabolic regulation; aging effects can interact with diabetes to accelerate the progression of diabetic complications (74). Additionally, older adults are more susceptible to hypoglycemia and its consequences such as falls and consequent fractures, cardiovascular events, and mortality (75–77). Therefore, older adults with DM can be prone to feel nervous, anxious, or on edge (i.e., GAD1 “Nervousness or anxiety”). This distinguishes them from other populations and warrants further validation in future studies.

People with comorbid depressive and anxiety symptoms tend to respond poorly to treatment, have a longer duration of illness, and experience poor prognoses (12). The results of this study found GAD1 “Nervousness or anxiety” and CESD4 “Everything was an effort” to be critical bridge symptoms, indicating their roles in the development and maintenance of concurrent depression and anxiety in older adults with DM. Bridge symptoms could facilitate the spread of activation of one mental disorder to another, thereby contributing to contagion between disorders, and providing a new perspective for explaining comorbidity (25). GAD1 “Nervousness or anxiety” was identified as the bridge symptom, indicating the role of anxiety in the development of depression. This finding is consistent with prior research that has found “Nervousness” to be the bridge symptom between depression and anxiety for nursing students (30, 78). Our results showed that GAD1 “Nervousness or anxiety” was positively linked to many anxiety symptoms, such as CESD10 “Sleep disturbances” and CESD3 “Feeling blue/depressed”. This is consistent with prior studies that showed that the edges between “Nervousness or anxiety” and “sad mood” and between “Nervousness or anxiety” and “Sleep difficulties” are bridge pathways between depression and anxiety (symptoms measured using the PHQ-9 and GAD-7, respectively) (28, 62). Similarly, we found the influential bridge symptom within the depression community to be CESD4 “Everything was an effort”, suggesting it has an important role in contagion from depression to anxiety. Specifically, CESD4 “Everything was an effort” had relatively strong and positive associations with GAD1 “Nervousness or anxiety” and GAD3 “Worry too much”. Our findings indicated that feeling that everything was an effort might increase the risk of anxiety symptoms such as nervousness/anxiety and worrying too much. However, no previous studies have reported finding that “Everything was an effort” was the bridge symptom, consequently, a direct comparison cannot be made, and hence this issue is worthy of further study. Although we adopted a cross-sectional design and thus causality cannot be inferred from our study, our findings provide preliminary insights into the hallmark bridge symptoms facilitating the comorbidity of depression and anxiety. Moreover, our findings are in accordance with prior longitudinal studies that demonstrated that anxiety and depression are reciprocal risk factors for one another: that is to say, anxiety symptoms can lead to depressive symptoms and vice versa (11, 79–82).

The prominent central and bridge symptoms that were identified in the depression-anxiety network have potential clinical implications. According to the theory of psychopathological network, interventions targeting important central symptoms may have the greatest effect in destroying the overall network and reducing the severity of the network as a whole, facilitating intervention and treatment (19, 58). This study thus provides guidance for intervention strategies and suggests that targeting the symptoms “Feeling blue/depressed”, “Nervousness or anxiety”, “Uncontrollable worry”, “Trouble relaxing”, and “Worry too much” may be conducive to the prevention and treatment of depression and anxiety. Similarly, deactivating important bridge symptoms can disrupt the connections between comorbid mental disorders and prevent the contagion of one disorder to another, thereby reducing comorbidity (25). Based on the results of the present study, the bridge symptoms “Nervousness or anxiety” and “Everything was an effort” are recommended as intervention targets for the prevention and reduction of comorbid depression and anxiety disorders. Cognitive behavior therapy (CBT) is an effective treatment that is commonly used in the prevention of and intervention for depressive and anxiety symptoms in people with DM (83–85). Our findings indicate that CBT strategies (e.g., cognitive restructuring and behavioral activation) focusing on the central symptoms and bridge symptoms may be of benefit for the prevention and treatment of depression and anxiety and reduce their comorbidity in older adults with DM, although this needs further empirical research.

The strengths of this study include its large sample size, the representative study sample, and the utilization of network analysis to visualize depressive and anxiety symptom structures in older Chinese adults with DM with stable results. However, this study also has several limitations that should be noted. First, due to the cross-sectional design of our study, we could not infer the direction of causality between depression and anxiety. For insights into the temporal relationships, longitudinal research is needed. Second, the depressive and anxiety symptoms were measured using self-report scales, which may induce recall bias and remind us to interpret the results cautiously. Third, the findings may have limited generalizability as our sample focused on older Chinese adults with DM, and it is not known how generalizable our findings are to other populations. The applicability of our results to other populations with DM or older adults with clinically diagnosed depression and/or anxiety also requires replication. Fourth, the network did not include covariates or confounders such as diabetes complications, individuals’ personality traits, and biological factors which should be considered in future studies. Fifth, the type of diabetes was not considered since it was not recorded in the CLHLS dataset we used. Although type 2 diabetes was predominant among the older adults, future studies should examine whether the type of diabetes has an effect on the network structure. Finally, the network structure constructed in this study only reflects group effects, meaning that it cannot capture idiographic individual-level processes of depression and anxiety.

## 5 Conclusion

This study presents the first application of symptom-level network analysis to investigate the depressive and anxiety symptoms of older Chinese adults with DM. The results revealed that “Feeling blue/depressed”, “Nervousness or anxiety”, “Uncontrollable worry”, “Trouble relaxing”, and “Worry too much” were the most central symptoms and that “Nervousness or anxiety” and “Everything was an effort” were the key bridge symptoms within the depression-anxiety network. These identified symptoms may be potentially effective targets for the prevention of depression and anxiety among at-risk older adults with DM and inform treatment strategies for those who have depression and anxiety.

## Data availability statement

Publicly available datasets were analyzed in this study. This data can be found here: https://opendata.pku.edu.cn/dataset.xhtml?persistentId=doi:10.18170/DVN/WBO7LK.

## Ethics statement

The studies involving humans were approved by Biomedical Ethics Committee, Peking University (IRB00001052–13074), and the Institutional Review Board, Duke University (Pro00062871). The studies were conducted in accordance with the local legislation and institutional requirements. The participants provided their written informed consent to participate in this study.

## Author contributions

YZ: Data curation, Formal analysis, Methodology, Writing – original draft, Writing – review & editing. YC: Formal analysis, Writing – original draft. YL: Writing – original draft. HL: Data curation, Software, Writing – review & editing. HH: Methodology, Visualization, Writing – review & editing. JS: Formal analysis, Investigation, Visualization, Writing – original draft. ZG: Data curation, Formal analysis, Resources, Writing – original draft. DM: Formal analysis, Funding acquisition, Project administration, Resources, Validation, Visualization, Writing – original draft, Writing – review & editing.
