# Supplementary material for: Network analysis of depressive and anxiety symptoms in older Chinese adults with diabetes mellitus
Source: Front Psychiatry. 2024 Jan 29;15:1328857. doi: 10.3389/fpsyt.2024.1328857 (PMC10859512; doi:10.3389/fpsyt.2024.1328857)
Supplement: Supplementary file 1 [file DataSheet_1.pdf]

Supplementary materials

Supplementary Table 1 The edge weights within the depression-anxiety network

	CESD1	CESD2	CSED3	CESD4	CESD5	CESD6	CESD7	CESD8	CESD9	CESD10	GAD1	GAD2	GAD3	GAD4	GAD5	GAD6	GAD7
CESD1	0																
CESD2	0.15	0															
CESD3	0.33	0.11	0														
CESD4	0.05	0.26	0.16	0													
CESD5	0	0	0.01	0.02	0												
CESD6	0.17	0.04	0.19	0.03	0.01	0											
CESD7	0.04	0	0.02	0.02	0.39	0	0										
CESD8	0.06	0.01	0.08	0.05	0	0.25	0.11	0									
CESD9	0	0.05	0.13	0.09	0.13	0.08	0.03	0.30	0								
CESD10	0.04	0	0	0.04	0.06	0	0.09	0.04	0	0							
GAD1	0	0.04	0.05	0.05	0.05	0.05	0	0.01	0.03	0.10	0						
GAD2	0	0.01	0	0.01	0.01	0	0	0.02	0.02	0	0.23	0					
GAD3	0.02	0.01	0.01	0.06	0	0.01	0	0	0	0	0.11	0.29	0				
GAD4	0.01	0	0	0	0	0.02	0.02	0	0.02	0.02	0.16	0.23	0.23	0			
GAD5	0	0	0	0	0	0.01	0	0	0	0	0.01	0.15	0.08	0.18	0		
GAD6	0.02	0	0.04	0.01	0	0	0	0	0.03	0	0.12	0	0.14	0.07	0.30	0	
GAD7	0	0.01	0	0	0	0	0	0.02	0	0	0.04	0.09	0.05	0.07	0.21	0.16	0

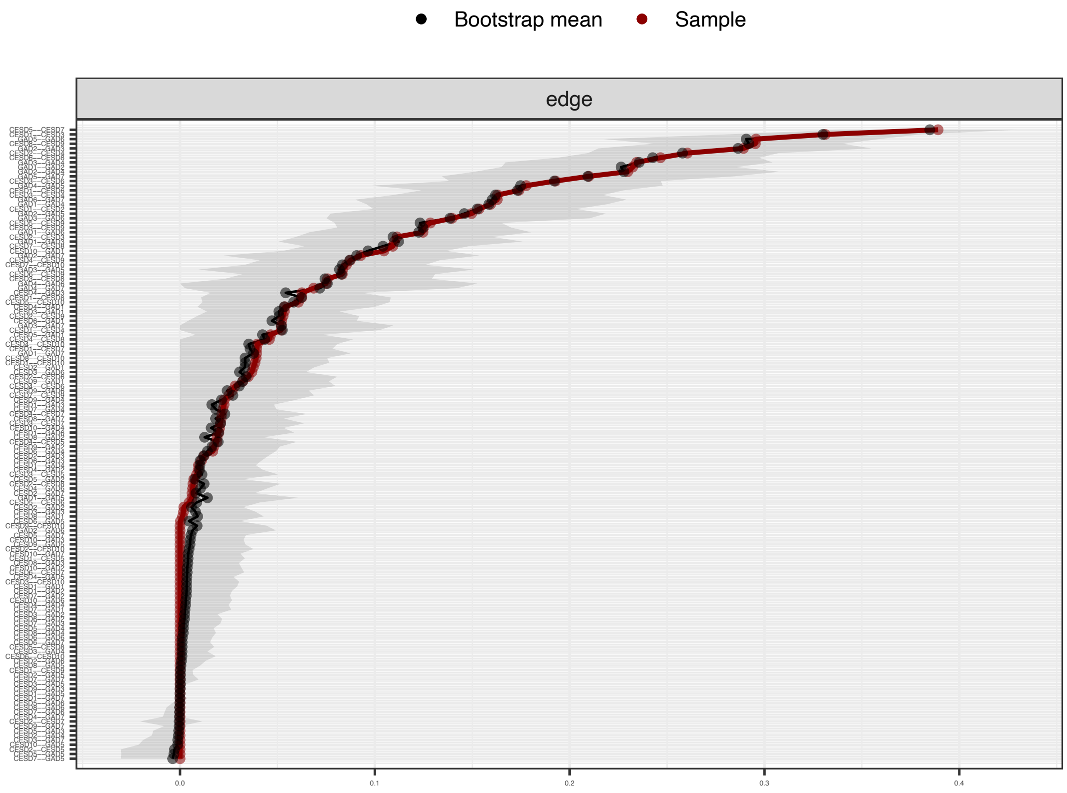

Supplementary Figure 1 Accuracy of edge weights in the network

Note: The red line depicts the sample edge weights, the black line represents bootstrap means, and the gray bar depicts the bootstrapped confidence interval.

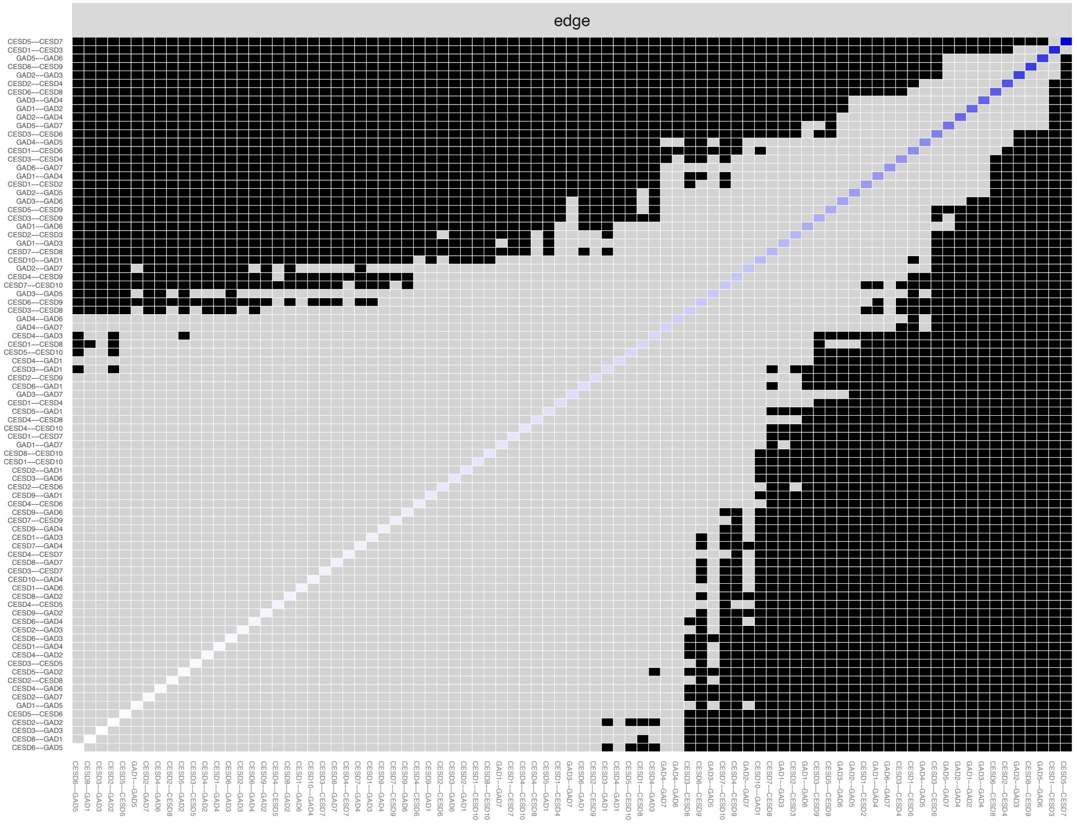

Supplementary Figure 2 Bootstrapped difference test for edge weights in the network

Note: Gray boxes indicate edge weights that do not differ significantly from one another, while black boxes indicate edge weights that do differ significantly. Blue boxes on the diagonal correspond to edge weights with positive correlations. All edge weights were positive.

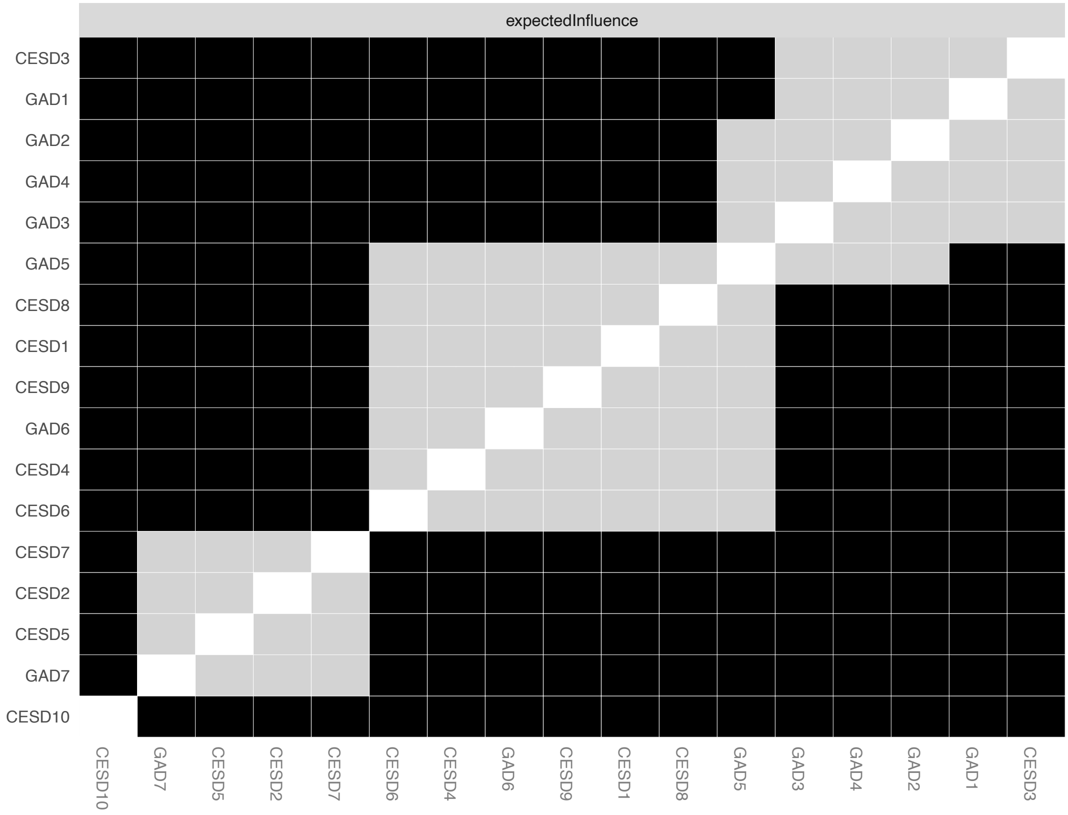

Supplementary Figure 3 Bootstrapped difference test for node expected influences in the network

Note: Gray boxes indicate node expected influences that do not differ significantly from one another, while black boxes indicate node expected influences that do differ significantly.

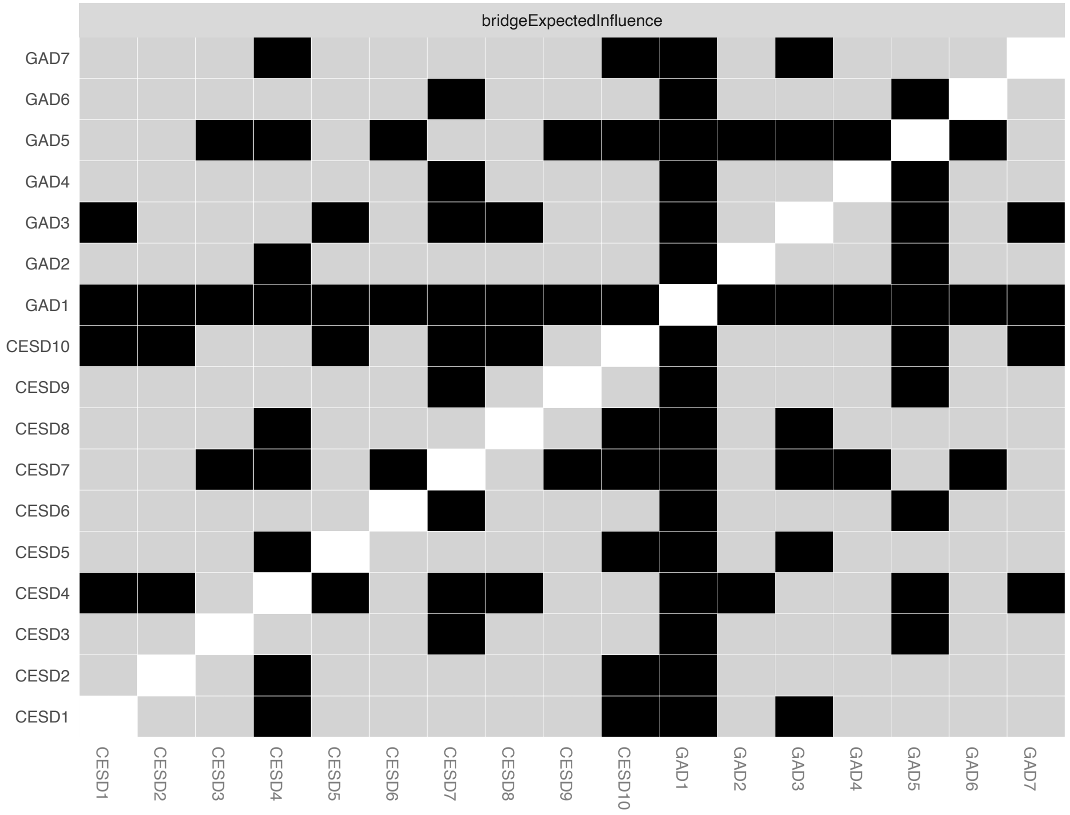

Supplementary Figure 4 Bootstrapped difference test for node bridge expected influences in the network

Note: Gray boxes indicate node bridge expected influences that do not differ significantly from one another, while black boxes indicate node bridge expected influences that do differ significantly.
